# Development of Salmonellosis as Affected by Bioactive Food Compounds

**DOI:** 10.3390/microorganisms7090364

**Published:** 2019-09-18

**Authors:** Ajay Kumar, Abimbola Allison, Monica Henry, Anita Scales, Aliyar Cyrus Fouladkhah

**Affiliations:** 1 Division of Gastroenterology, Hepatology and Nutrition, Department of Pediatrics, University of Virginia School of Medicine, Charlottesville, VA 22908, USA; 2 Public Health Microbiology Laboratory, Tennessee State University, Nashville, TN 37209, USA; abimbolaallison20@gmail.com (A.A.); mhenry3@my.tnstate.edu (M.H.); ascales3@my.tnstate.edu (A.S.); 3 Cooperative Extension Program, Tennessee State University, Nashville, TN 37209, USA

**Keywords:** dietary bioactive components, salmonellosis, bile acids, epithelial barrier, gut microbiota

## Abstract

Infections caused by *Salmonella* serovars are the leading cause of foodborne hospitalizations and deaths in Americans, extensively prevalent worldwide, and pose a considerable financial burden on public health infrastructure and private manufacturing. While a comprehensive review is lacking for delineating the role of dietary components on prevention of Salmonellosis, evidence for the role of diet for preventing the infection and management of Salmonellosis symptoms is increasing. The current study is an evaluation of preclinical and clinical studies and their underlying mechanisms to elaborate the efficacy of bioactive dietary components for augmenting the prevention of *Salmonella* infection. Studies investigating dietary components such as fibers, fatty acids, amino acids, vitamins, minerals, phenolic compounds, and probiotics exhibited efficacy of dietary compounds against Salmonellosis through manipulation of host bile acids, mucin, epithelial barrier, innate and adaptive immunity and gut microbiota as well as impacting the cellular signaling cascades of the pathogen. Pre-clinical studies investigating synergism and/or antagonistic activities of various bioactive compounds, additional randomized clinical trials, if not curtailed by lack of equipoise and ethical concerns, and well-planned epidemiological studies could augment the development of a validated and evidence-based guideline for mitigating the public health burden of human Salmonellosis through dietary compounds.

## 1. Introduction

Despite increased awareness and development of treatments such as antimicrobial interventions in manufacturing and antibiotic therapies in healthcare facilities for over a hundred years, *Salmonella* serovars are still a major concern in infectious diseases related premature morbidity and mortality [1,2,3,4]. Various serovars of *Salmonella* are the leading cause of foodborne hospitalizations and deaths in Americans causing over one million, about 20,000, and 378 annual illness, hospitalization, and deaths episodes, respectively [5]. Non-typhoidal *Salmonella* serovars are also the leading agent among most common foodborne infectious diseases, responsible for highest number (32,900 years) of disability adjusted life year (DALY), annually [6]. National Antimicrobial Resistance Monitoring System (NARMS) and other epidemiological sampling also reveal a widespread presence of multiple drug resistance (MDR) phenotypes of the pathogen in various facilities—as an example, 0.6% of ground meat samples may harbor MDR *Salmonella* [7] with approximately 7% of them displaying MDR-AmpC phenotype [8]. As such, the U.S. Department of Health and Human Services had categorized non-typhoidal *Salmonella* as a “serious threat” to the public health [9].

Data from world population also indicate that the pathogen is one of the leading causes of deaths associated with diarrheal diseases globally, with estimated 3.4 million cases (invasive non-typhoidal *Salmonella* serovars) and over 600,000 deaths annually [10,11]. The bacterium is a Gram-negative organism with a complicated and evolving nomenclature, currently consist of two species, at least six sub-species, and over 2500 serovars [12].

Changes in production and manufacturing practices, increased international commerce and travel, increased proportion of at-risk populations for infectious diseases, and changes in population’s eating habits during last few decades had contributed to increased incidences of *Salmonella* infections [13,14]. *Salmonella* serovars induce acute inflammation in the intestinal track after infection and utilizes the environment to further proliferate and colonize [15,16,17]. Colonization resistance against *Salmonella* is modulated by gut microflora, intestinal immunity, epithelium, and quality and quantity of digestive fluids. Various food components have been shown to modulate these factors and could be a potential intervention for reducing the likelihood of enteric infections.

Over the past 20 years, role of the dietary agents in shaping immunity against enteric infections has becoming increasingly evident [18,19,20,21,22] and piqued the interest in nutritional interventions for enteric infections. Several dietary components ranging from polyphenolic compounds, fibers, micronutrients, fatty acids, peptides, and carbohydrates of plant and animal origin had been shown efficacious against *Salmonella* serovars in various experimental models [22,23,24,25,26,27,28,29,30,31,32]. These associations are the result of an array of potential biochemical pathways, very complex and dynamic in nature, including interactions among dietary components, gut epithelium, digestive system, immune system and gut microbiota as affected by various seasons [33,34,35,36,37]. Better understanding of these underlying mechanisms could reduce *Salmonella* prevalence in the food chain though modifications in food animal diets. It could further reduce the public health burden of non-typhoidal *Salmonella* serovars by mitigating severe symptoms and reducing the pathogen DALY and mortality rate in healthcare facilities for Salmonellosis patients. Hence, the current work is a review of *Salmonella* infection studies as affected by various dietary components with discussions of the mechanisms of action and types of preclinical, animal models, and clinical studies employed.

## 2. Current Status of Knowledge

### 2.1. Effect of Dietary Components against Salmonella: In-Vitro Models

**Figure 1:**
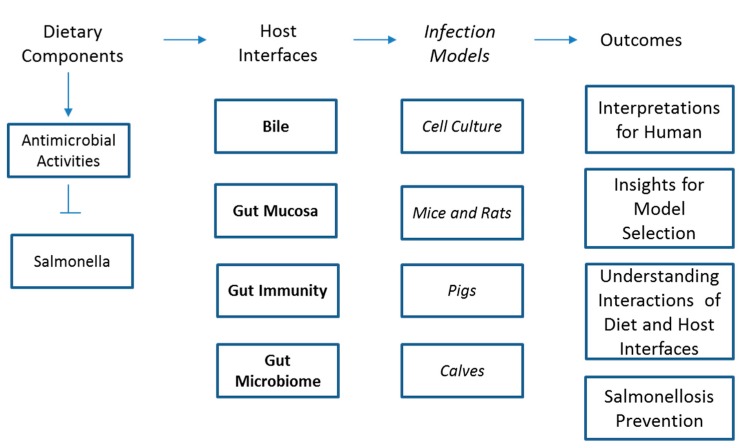
Relationships between dietary bioactive components and *Salmonella* infection. Dietary bioactive components such as fiber, amino acids, vitamins and minerals, fatty acids, and polyphenols improve the gut epithelium, microbiota, and immunity that may eventually lead to increased resistance to *Salmonella* infection.

Dietary components may prevent infection outcome by directly affecting the pathogen multiplication and virulence [7,38,39] or by modulating host response to the pathogens [26,37]. To test the direct effects of dietary components on pathogens, researchers have used food extracts or dietary bioactive components on *Salmonella* cultures. Summary of potential relationships between dietary components and *Salmonella* infections are presented in Figure 1. Following treatment of dietary components, multiplication, and gene expression for virulence and motility could be measured. These models are comparatively less expensive and less cumbersome to assess the efficacy of dietary components for *Salmonella* infection. For instance, several essential oils were added to *Salmonella* growth media at various doses and *Salmonella* multiplication was compared with untreated controls [38]. Among 28 tested essential oils, *Origanum heracleoticum, Cinnamomum cassia, Corydothymus capitatus, Satureja montana*, and *Cinnamomum verum* were particularly effective against *Salmonella* Typhimurium [38]. Citrus flavonoids were similarly evaluated on *Salmonella* virulence gene expression [39]. In the study, Naringenin, a flavanone present in grapefruit, repressed 24 genes in pathogenicity island of *Salmonella* Typhimurium LT2 and further down-regulated 17 genes associated with the pathogen motility [39]. Most recent studies also reveal similar trends, for example, various essential oils extracted from *Aloysia triphylla, Cinnamomum zeylanicum, Cymbopogon citratus, Litsea cubeba, Mentha piperita*, and *Syzygium aromaticum* had been shown to be efficacious against *Salmonella* serovars during in vitro challenge studies [40].

It is noteworthy that aforementioned studies are conducted without host interaction and interpretation and generalization of the results should be drawn with caution and after further investigations in presence of host cells.

Orally infected *Salmonella* can enter circulation through various routes. It can invade several phagocytic and non-phagocytic cells depending upon serotype. In the murine model, *Salmonella* invades both phagocytic and epithelial non-phagocytic cell types. Hence, in vitro models of *Salmonella* entry have been developed to assess the effect of a test compound on a host. The *Salmonella* entry model could reveal the mechanism of action of a test compound on an organism. Several human and mouse cell lines such as Caco-2 [41] and RAW264.7 have been used in the literature to test efficacy of the compounds against *Salmonella* entry. For example, secretory immunoglobulin A (SIgA) was demonstrated to be a potent inhibitor for *Salmonella* Typhimurium entry into polarized monolayers of HeLa cells [42].

*Salmonella* contains the pathogenicity islands for the secretion of effector molecules to infect the target cells [43]. The molecules released by these secretory systems change the host cell cytoskeleton to facilitate *Salmonella* entry. The in vitro *Salmonella* entry models are impactful in studying the effects of dietary components on *Salmonella* as well as on host cells. However, these studies do not represent involvement of all host cell types that are simultaneously present in gastrointestinal area of humans. Dietary components could affect *Salmonella* virulence by affecting secretory systems or by competing with *Salmonella* for the receptors on host cells [39]. Host cells can also release the cytokines in response to the dietary components that can affect *Salmonella* virulence or motility. Therefore, in vitro models of *Salmonella* infection can have great implications for assessing mechanisms of actions by the dietary components.

### 2.2. Summary of Effect of Dietary Components on Salmonella Infection in Rodent Models

The fecal shedding of *Salmonella*, tissue colonization, local and systemic inflammatory changes, survival and weight reduction are the major observable changes associated with *Salmonella* infections in rodents. Bovee-Oudenhoven et al. showed reduced *Salmonella* fecal shedding when fructooligosaccharides were fed to the male Wistar rats as compared to the cellulose-fed group after 2 weeks of dietary intervention [44]. Furthermore, dietary fructooligosaccharides increased fecal *Lactobacilli* count and increased the translocation of *Salmonella* to the liver and spleen with an increase in fecal mucin as compared to cellulose fed rats [19]. The author concluded that dietary fructooligosaccharides decreased *Salmonella* colonization but increased the translocation potentially due to irritation of mucosal membrane. Some of the mice strains succumb easily to *Salmonella* infection and hence survival rate is the primary indicator of the dietary efficacy against infection. Hitchins et al. showed that feeding of freeze dried yoghurt to male weanling Sprague-Dawley rats increased overall survival rate and weight of the animals after intraperitoneal *Salmonella* challenge as compared to rats fed on milk diet for 1 week [45]. Similarly, dietary feeding of Herba Pogostemonis extract to Balb/c mice increased the overall survival rate as compared to control diet fed animals after intraperitoneal *Salmonella* challenge [46]. Feeding of Herba Pogostemonis (*Pogostemon cablin* Bantham extract) also reduced *Salmonella* liver damage as compared to control diet fed animals [46]. Recent studies similarly show association among various bioactive food compounds and prevention of Salmonellosis. Supplementing the diet of albino rats with olive oil, as an example, had been shown to have efficacy against *Salmonella* Typhi as a natural antimicrobial and non-toxic immune modulator [47]. These studies show that there are measurable markers for *Salmonella* infections in rodents and they can be used as a model to mimic *Salmonella* infections in human host.

Both foodborne pathogens and dietary components pass through the stomach acid, when ingested. Hence, gastric acidity is one of the important factors in determining stability of enteric pathogens. In a randomized controlled clinical trial, gastric hypochlorhydria (low hydrochloric acid) was found to be associated with increased *Salmonella* infections [48]. This hypothesis was also confirmed in the rodent model of *Salmonella* infection. Tennant et al. [49] showed that treatment of mice with antacids resulted in the decreased infectious dose of *Salmonella* as compared to normal mice.

Similar results were also observed in a constitutively hypochlorhydric mice (proton pump mutation) as compared to the normal mice [49]. Additionally, gastric pH not only affects the survival of pathogens but also affects digestion and absorption of foods. Lucas et al. showed that an increase in pH from 1.5 to 2.5 reduced digestion of the kiwifruit peptides [50]. Gastric pH also modulates absorption of micronutrients such as zinc. Henderson et al. observed higher plasma zinc levels in the young healthy volunteers at low pH as compared to plasma level in higher gastric pH volunteers [51]. The gastric pH is considerably different across species. For instance, mean gastric pH in mice is 3.1–4.5 and in rats ranges from 3.2 to 3.9, whereas in the humans it is 1.5–3.5. In addition to gastric pH, intestinal pH is also different in rodents as compared to humans. Mice and rats have a mean intestinal pH of 5.2 and 6.6, respectively, as compared to 7.2 in humans [52]. These studies show that gastric and intestinal pH could potentially affect bioactivity of dietary components and should be considered as one of the important factors in selecting a study model.

In rodents and humans, several disease symptoms can be confounding due to the differences in their anatomy and physiology. For example, in the non-typhoidal salmonellosis, vomiting and diarrhea are the main symptoms in humans. However, anatomically mice cannot vomit and due to this reason, the assessment of diarrhea could be very difficult in mice. In these cases, it becomes harder to translate the finding into clinical applications. Hence, these limitations of rodent models should be taken into consideration while interpreting the results from the dietary intervention studies for *Salmonella* infections in the rodent models for application in human clinical trials.

### 2.3. Summary of Effect of Dietary Components on Salmonella Infection in Pig Models

Pigs have been used in several studies involving dietary interventions [53]. Pigs have many more similarities to the human gastrointestinal tract as compared to rodents. Humans and pigs are similar in the body composition, cardiovascular, renal, nutritional, immunological, metabolic, and gastrointestinal aspects [53]. As such, several studies have been conducted in pig models of *Salmonella* infection interactions with dietary interventions. Michiels et al. demonstrated that supplementation of a mixture of formic, sorbic, and benzoic acid to the piglets for 35 days, significantly reduced the *Salmonella* fecal shedding as compared to the control group after oral challenge [54]. Dietary organic acids increase fecal cytotoxicity to *Salmonella*, but the effect can be dependent upon the environmental temperature. Rajtak et al. exhibited that supplementation of a pig diet with organic acid (Potassium- diformate) reduced the survival of *Salmonella* in pig feces when incubated at 22 °C but not at 4 °C [55]. Boyen et al. fed the supplemented diet with the coated butyric acid (2 g/kg of diet) to the pigs for 12 days and orally challenged the animals with *Salmonella* [56]. Fecal shedding of *Salmonella* was decreased in the coated butyric acids fed animals as compared to the un-coated group. It was hypothesized that coating prevents the degradation of fatty acids in the intestinal tract [56]. Dietary supplements also reduced inflammation after *Salmonella* infection in pigs. Chen et al. supplemented the pig diet with arginine (0.5%) for 1 week and infected the pigs intramuscularly with *Salmonella* [57]. Effects of various essential oils have been similarly reviewed by Omonijo et al. as effective antimicrobials in Swine production [58].

Fecal *Salmonella* shedding is one of the distinctive biomarkers of *Salmonella* infection in pig models. However, *Salmonella* colonization patterns are different in pigs as compared to humans. For instance, *Salmonella* Typhimurium has been observed to colonize in tonsils and respiratory tissues of infected pigs [59], whereas in humans, it does not colonize at those sites. The pig stomach is 2–3 times larger compared to humans [52], this anatomical difference may have impacts on *Salmonella* survival and digestibility of dietary components. Pig cecum is also several folds larger than the human cecum and may have implications in the *Salmonella* colonization [52]. In humans, stomach pH before eating is around 5, however, in pigs it is below 2. Consequently, pigs release a much greater extent of bile in the duodenum as compared to humans. Due to antimicrobial activities, bile could impact colonization of *Salmonella* in the proximal small intestine. Additionally, it can modulate digestion and absorption of the dietary components. Besides these differences, pigs are different in gastrointestinal thickness of mucus, and gastrointestinal motility and transit, as compared to humans. The distal small intestine of pigs contains a larger number of microbes as compared to humans and can degrade some carbohydrates with low digestibility compared to humans [60]. Hence, similar dietary interventions in pigs and humans may exhibit different potential. The pig immune system also differs from humans, however, implications of this difference have not been studied in regard to enteric infections. For instance, the gut of neonate piglets completely lacks leukocytes whereas human infants have a few leukocytes at birth [61]. Pig intestine contains a larger number of Peyer’s patches as compared to humans throughout the intestine [61].

### 2.4. Summary of Effect of Dietary Components on Salmonella Infection in Calf Models

Although there are appreciable differences between monogasters and ruminants, calves develop very similar clinical and pathological features such as diarrhea and enteritis to human, hence, calves are considered one of most reliable models to mimic the human non-typhoidal salmonellosis [36]. These similarities have been also discussed by Higginson et al. [62].

After *Salmonella* infection, the calves show similar clinical symptoms as humans such as fever, diarrhea, anorexia and dehydration and the intestinal pathological changes [63]. Hill et al. revealed that feeding of a commercially available blend of butyric acid, coconut oil, and flax oil to the male Holstein calves for 28 days altered the inflammatory response to intraperitoneal *Salmonella* toxoid as compared to the control group [64]. The dietary blend reduced hyperthermia, hypophagia, and serum TNF-α but increased the IL-4 as compared to the control group [64].

Despite above-mentioned similarities, calves also exhibit significant anatomical and physiological differences in the digestive system relative to humans. A ruminant’s stomach is four chambered and contains a large number of microflora that digests fibers, especially cellulose which remain undigested in humans. Sugars are fermented in ruminant stomach and as a result several volatile fatty acids are produced [65]. Most of the carbohydrates are converted into volatile fatty acids and a very small proportion of carbohydrates are absorbed as glucose. Additionally, the ruminant microflora differs from the human gut microflora to a great extent [66]. Hence, the same dietary components may produce different metabolites and physiological effects as compared to humans. Logistically, calves need a large amount of food and it is very expensive to conduct the dietary experimental studies in this model.

### 2.5. Summary of Dietary Interventions for Salmonella Infection in Humans

A variety of *Salmonella* serovars infect humans. Epidemiological studies have shown that typhoidal and non-typhoidal salmonellosis are the predominant types of infections [67]. Salmonellosis is clinically prognosed by headache, diarrhea, constipation, abdominal pain, chills, loss of appetite, and fever with an incubation time varying from hours to several days [68,69]. Typhoidal salmonellosis is less prevalent in the United States as compared to other developing countries [67]. In contrast, non-typhoidal salmonellosis presents a major and persisting public health challenge in North America. From 1998 to 2017, over 2600 single or multi-state non-typhoidal *Salmonella* outbreaks have occurred in the United States associated with animal and plant based foods [70]. Symptoms could be self-limiting, lasting for 1 week without treatment but could also lead to serious complications if left untreated, especially in immunocompromised subjects and those in at-risk populations [69]. Antimicrobial therapy is the first choice of treatment in persistent human salmonellosis. However, as discussed in the introduction section, the problem of drug resistance has become more prevalent due to extensive therapeutic use of antibiotics in healthcare facilities and subtherapeutic doses during animal food production [71]. Hence, dietary prophylactic interventions could be further utilized for prevention and alleviating symptoms of *Salmonella* infections. A few dietary prophylactic studies have been conducted in children for prevention of *Salmonella* infections. Stool frequency, vomiting, and *Salmonella* fecal shedding are the parameters measured in these clinical trials. Several other disease conditions also affect the incidence of *Salmonella* infections. Di Cagno et al. revealed that administration of gluten free diets in children with celiac disease did not reduce *Salmonella* shedding from stool as compared to healthy children [72]. Other dietary interventions are effective in reducing *Salmonella* infection. Lara et al. showed that feeding of dairy products containing probiotic mixtures of various strains of *Lactobacillus* to healthy children for 6 weeks decreased *Salmonella* serovars adhesion to the intestinal mucin [73]. Dietary interventions can also reduce frequency of stool and vomiting in *Salmonella* infected children. Rabbani et al. revealed that feeding of cooked banana for 1 week in children having persistent diarrhea, reduced frequency of the stool and vomiting as compared to children fed only with rice diet [74]. In another clinical trial in children, fermented food (lactic-acid fermented cereal gruel) was fed to healthy children three times a day for 2 weeks. After 2 weeks of feeding, stool swabs were taken from the treated and non-treated groups and analyzed for the presence of enteropathogenic bacteria including *Salmonella*. The fermented food reduced the presence of enteropathogenic bacteria as compared to the control diet [75]. These studies show that dietary interventions can be effective in the management of diarrheal diseases. However, there are several constraints in conducting dietary studies in *Salmonella* infections in humans that are prophylactic in nature. In addition to clinical equipoise, the major issues in conducting human clinical trials are time, cost, availability of appropriate stool and serum biomarkers and overall patient compliance and ethics. In presence of these curtailments, a dietary intervention could be pre-clinically evaluated in a relevant animal model to predict the safety and efficacy of the compound prior to administration in clinical trials [76].

It is noteworthy that bioactive compounds and probiotic diet might have a positive effect on colonization of *Salmonella* serovars in gastrointestinal area. As an example, a probiotic diet containing *Enterococcus* spp. could lead to increased fecal excreting and colonization of *Salmonella* in organs of piglets [77]. The current study is limited to discussing the literature that demonstrates antagonistic efficacy against colonization of *Salmonella* serovars, rather than those enhancing proliferation of the pathogen. Table 1 summarizes the pros and cons of *Salmonella* models discussed in the current study.

## 3. Potential Mechanisms of Protection against *Salmonella* Infections

### 3.1. Alteration in Bile Quality and Quantity

Bile is an important digestive fluid synthesized by the liver of many vertebrates. Bile plays a role in digestion of fats in small intestine by emulsification, micelle formation. As a result, absorption of fat-soluble vitamins such as vitamin A, D, E, and K is also increased in the presence of bile. Bile is stored in the gall bladder and released into the duodenum after receiving stimuli in the form of semi-digested fats and proteins from stomach. After digestion of fats, the majority of the bile is reabsorbed in terminal ileum. Cholycystokinin and secritin hormones in the gut control this process. Bile is alkaline and composed of phospholipids, bile acids, and surfactants. In the duodenum alkaline pH neutralizes stomach acid [78].

In addition to digestive role of bile, it exhibits an antimicrobial role against gastrointestinal pathogens [79]. Both bile quality and bile quantity may determine the multiplication of enteric pathogen [80]. Bile salts have been shown to act as antimicrobials especially on *Salmonella* and other enteric infections [81,82]. Different dietary fibers have been shown to affect bile composition to different extent and to improve colonization resistance against enteric pathogens [83]. Inagaki et al. showed that bile acids induces genes involved in enteroprotection by inhibiting pathogenic overgrowth and mucosal injury in the ileum in a mouse model of infection [84]. Diet consists of several compounds of plant and animal origin and hence considered as a multi-targeting intervention for prevention of enteric infection. Xu et al. [85] showed that consumption of dietary medium chain fatty acids increased fecal bile acids (cholic acid) significantly as compared to control group in C57BL/6J Mice. Kollanoor et al. demonstrated that feeding of Caprylic acid (a medium chain fatty acid) to poultry significantly reduced *Salmonella* infection in the intestine as well in organs therapeutically [83]. Further, in vitro study in hepatocytes showed that addition of medium chain fatty acids in culture media enhances cell surface expression and transport capacity of bile salt export pump (BSEP/ABCB11) [86]. Costarelli et al. compared diets containing different fatty acids in healthy premenopausal women and found that dietary linoleate increased postparandial plasma bile acid and cholycytokinin as compared to low fat diet [87]. Dietary fish oil increased fecal bile acids in a rodent model without increased gene expression for bile synthesis in the liver [88]. This study suggests although not all fatty acids increase bile acid synthesis in the liver, some could reduce bile absorption in the ileum. Studies have further exhibited that the change in bile acid release alters pH of the intestine and affects *Salmonella* adherence and survival. Several *Salmonella* genes are affected in the presence or absence of bile. Both bile quality and quantity have been shown repress *Salmonella* virulence in gut environment in in vivo models [89,90,91]. Antunes et al. [92] showed that *Salmonella* could multiply in the gall bladder of susceptible mice and causes typhoid. Bile acids exert antimicrobial actions on pathogens by virtue of their detergent properties. Cholic and deoxycholic acids in bile can damage bacterial DNA [79].

Dietary factors such as fiber may bind to bile acids and reduce reabsorption in colon [93]. Oat bran, pectin, and guar gum have been shown to increase bile acids in fecal matter [94,95,96]. Reduction in reabsorption of bile acids in the large intestine modulates the gut hormone feedback system and stimulates the liver to synthesize more bile acids [78]. This process could reduce alkalinity of the small intestine, and may increase gut motility, making the gut environment unfit for *Salmonella* infection [97].

### 3.2. Gut Mucosa

In order to reach epithelium, *Salmonella* needs to cross luminal barriers. Intestinal mucous is the first line of defense to *Salmonella* in the small intestine of rodents and humans [98]. Mucus in the small intestine is single layered and loosely attached to epithelium as compared to double-layered mucus of colon. Mucous is made up of secretory proteins called mucins and the predominant mucin in small intestine is Muc2 [99]. Abnormalities in mucous layers, underproduction of Muc2 by goblet cells and mutated Muc2 results in elevated risk for bacterial infection [100]. A study shows that during *Salmonella* infection, the mucin layer is disrupted and *Salmonella* obtain access to epithelium [101].

Various components of diet have been shown to upregulate expression of Muc2 in intestinal cells. Willemsen et al. showed that treatment of intestinal epithelial and fibroblast co-culture with short chain fatty acids significantly increased expression of Muc2 [102]. Ingestion of dietary fibers (soluble and insoluble) has been shown to increases proliferation of goblet cells and sialylated mucin in the small intestine of rats [103]. In another study, feeding of inulin/fructans in a rodent trial significantly increases mucous layer thickness in the colon and increases the number of goblet cells in crypts of distal jejunum as compared to control diet [104]. Morita et al. similarly exhibited that intake of dietary resistant starch in rodents reduces endotoxin influx from intestinal tissue and hypothesized that it could be partially due to alterations in mucosal barrier functions [105].

### 3.3. Antimicrobial Activities

After crossing the mucin layer in the gut, enteric pathogens need to penetrate epithelial layer in order to infect the organism. Human gut epithelia consist of a monolayer of epithelial cells. It separates the gut lumen from the lamina propria. Intestinal epithelial cellular junctions affect intestinal permeability as well as transcytosis capacity of individual cells. Strong cellular junctions are necessary to avoid the invasion of pathogens through epithelium. *Salmonella* can breach the epithelial barrier by employing para-cellular and trans-cellular mechanisms, including actin cytoskeleton of the epithelial cells and the secretion of the effector molecules [30].

Dietary components have been discussed in the past as factors to modulate the epithelial barrier [106]. Diet can have both positive and negative impacts on epithelial integrity. Liu et al. showed that when a high grain diet was fed to male goats, it resulted in the disruption of the ruminal epithelium as measured by the presence of systemic lipopolysacharide (LPS) [107]. However, diet can also impact epithelial integrity positively. In a study by Nofrarias et al., pigs were fed resistant starch for 97 days and consequently increased hypertrophy, reduced apoptosis in the crypts, lymphoid nodules in the colon, and increased mucin sulfuration were observed. These changes promoted epithelial protection compared to the control dietary group containing digestible starch [108]. Dietary components can also modulate the epithelial proteins such as occludins that secure junctions between the adjacent cells in the gut epithelium. Enteric pathogenic bacteria secrete LPS that causes inflammation and escalates loss of protein occludin that decreases the barrier function of epithelium. Park et al. showed in a rodent trial that dietary administration of gangliosides (a lipid) prevents LPS induced degradation of the occludin and reduces the total nitric oxide in the gut mucosa [109]. An in vitro study with Caco-2 cells demonstrated that addition of quercetin (a flavonoid) induces expression of zonula occludens-2, occludin, and claudin-1 and claudin-4 as compared to the control group [110]. All of these proteins play an important part in maintaining epithelial integrity. *Salmonella* entry into epithelial cells can result in epithelial necrosis and apoptosis. Int-407 cell line (human intestinal cell line) showed a significantly lesser extent of necrosis and apoptosis during *Salmonella* infection when treated with sterols and fatty acids found in the root extract of *Hemidusmus indicus* as compared to an untreated cell line [111]. Hence, protection of the epithelium can be considered an important target of dietary interventions in *Salmonella* infections.

### 3.4. Gut Microbiome

The gut contains more than a trillion symbiotic bacteria that play a major role in developing immunity as well as resistance against enteric infections. Initially it was hypothesized that the genetic factors were responsible for susceptible and resistant mouse strains against the enteric infections. However, currently literature delineates that the genetic factors are only one of the determinants of composition and structure of the gut microflora. As an example, Willing et al. successfully transferred the microbiota from resistant to susceptible mice and observed a delayed colonization of *Citrobacter rodentium* and mortality in susceptible strain [112]. In the same study, native gut microbiota of resistant mice was depleted by oral streptomycin (20 mg) 24 h prior to transplantation and replaced by the microbiota from susceptible mice. As a result, the oral antibiotic treatment reduced the innate defenses and a severe infection pathology was observed as compared to mice in control group. This experiment demonstrates that gut microbiota plays an important role in fighting the infection [112]. Similarly, mice were given a combination of antibiotics (Streptomycin, Vancomycin, Ampicillin, Neomycin, and Metronidazole) for 1 week in drinking water and later orally challenged with *Salmonella* Typhimurium 14028. The mice on the antibiotics showed a significantly higher number of *Salmonella* DNA in the cecum and large intestine as compared to control mice group [113]. The gut microbiota may affect enteric infections by modulating the intestinal immunity or by the direct competition. Symbiotic gut microbiota competes with pathogens for the nutrients such as iron and carbon sources [114]. Stelter et al. showed that *Salmonella*-induced mucosal lactins kills symbiotic gut microflora and then *Salmonella* takes advantage of this process for survival in gastrointestinal tract [115]. *Salmonella* induces acute inflammation in mice and neutrophils are recruited at the site of infection. Gill et al. showed that neutrophil elastases can shift mice gut microbiota and increase *Salmonella* colonization, while neutralization of neutrophil elastases decrease colonization of *Salmonella* [116]. These studies show that gut microbiota play an important role in protection from *Salmonella* infections and modulation of gut microflora for prevention of enteric infections warrants further studies.

Given the role of gut microbiota in protection against *Salmonella*, several studies have been conducted to test effects of dairy and native gut probiotics on *Salmonella* colonization. Probiotics are the microorganisms that induce health benefits when consumed in effective doses. *Lactobacillus* and *Streptococcus* are two widely studied categories of probiotics and their effectiveness against *Salmonella* is articulated by Castillo et al. [117]. *Lactobacillus rhamnosus* has been shown to reduce *Salmonella* adhesion to epithelial cells in in vitro model of *Salmonella* infection [118]. Probiotics not only compete with *Salmonella* for nutrients but also enhance protective immunity against the pathogen. Castillo et al. showed that oral administration of *Lactobacillus* in mice changes cytokine production and Toll Like Receptor (TLR) expression that is protective for mice against *Salmonella* infection [119]. Moreover, probiotics such as *Bifidobacterium* can directly affect virulence of *Salmonella* by releasing the molecules that down-regulate the expression of pathogenicity islands 1 and 2 [120]. Hence, *Lactobacillus* and *Bifidobacterium* have emerged as potential contributors for protection against enteric infections such as *Salmonella* serovars.

Diet is a major factor in the establishment of gut microbiome. As an example, previous studies exhibit that a change of diet from low-fat, high plant-based polysaccharide to the high-fat, and high simple sugar diet, could change structure of the gut microbiota very rapidly [121]. A shift of low-fat diet to the Western diet also changes metabolic pathways and modulates gene expression in gut microbiome [122]. Humanized mice (mice transplanted with human gut microflora) when fed a Western-type diet, showed an increased adiposity and this trait was transmissible through the transplantation of the gut microbiota in other mice [122]. Diet could also modulate gut microbiota directly by providing prebiotics—many studies have exhibited the efficacy of the prebiotics such as dietary fiber, fatty acids, and polyphenols for a shift in gut microflora [123,124].

### 3.5. Gut Immunity

The immune system of the gastrointestinal tract is the largest segment of the mammalian immune system. The gut encounters massive amounts of pathogens and dietary antigens that need to be neutralized. These functions emphasize the importance of gut immune system. The mucosal immune system is equipped with innate and adaptive immune defense mechanisms. Innate immunity provides the first line of defense against pathogens. The major players of the innate immune defense are macrophages, monocytes, neutrophils, epithelial cells, natural killer (NK) cells, and dendritic cells (DCs) [125]. Dendritic cells, macrophages, and epithelial cells are also termed as antigen presenting cells (APCs) because of their capacity of processing and presenting foreign antigens to other cells. APCs have a series of receptors called Pattern Recognition Receptors (PRRs) on their surfaces such as TLRs and Nod Like Receptors (NODs) to recognize the pathogens [126]. These receptors recognize motifs on pathogens known as the Pathogen Associated Molecular Patterns (PAMPs) [127]. The innate immune cells release inflammatory cytokines and mediators after sensing the PAMPs [128]. However, if innate immunity fails to resolve the inflammation and eliminate pathogen, adaptive immunity enters this process. In the gut adaptive immune system, the predominant response is antibody mediated and is represented by the Immunoglobulin A (IgA) [129]. The IgA is chiefly produced by the B cells in the intestinal mucosa triggered by anti-inflammatory cytokines such as TGF-β and IL-10 [130]. Hence, both innate and adaptive immune responses are required in the protection against infection and depends upon type of pathogen.

The role of the gut immune system in protection from enteric infections has been studied intensely [131,132,133]. Primary *Salmonella* infection increases interferon gamma (IFN-γ), tumor necrosis factor alpha (TNF-α), and interleukin 12 (IL-12) in circulation and in local tissues [133,134,135]. Major sources of IFN-γ and TNF-α are neutrophils and macrophages [136]. IL-12 is a cytokine induced in response to several bacteria and mediates onset of the Th1 protective response. Natural killer T (NKT) cells produce IFN-γ in response to IL-12 [137]. Infected macrophages also interact with NK cells in order to produce IFN-γ in humans [138]. Even though initial innate immune response restricts infection to a certain extent, it fails to inhibit multiplication of pathogens in deeper tissues. Hence, immune response is switched to adaptive response after some time and is achieved mainly by induction of CD4+ T cells, CD8+ T cells, and B cells [139]. In experimental models, depletion of CD4+ T cells had a more pronounced effect on protection from *Salmonella* as compared to CD8+ T cells. However, underlying mechanisms are not clear. The second major adaptive response to *Salmonella* is induction of the B cells to produce antibodies such as IgA. The antibodies bind *Salmonella* and prevent entry into deeper tissues. Administration of B cell hybridoma producing *Salmonella* specific IgA has been shown to prevent oral *Salmonella* infection in the mice [140]. These studies exhibited the potentially appreciable role of bioactive compounds for augmenting host immunity against *Salmonella* infections.

Dietary components such as dietary fiber and prebiotics manipulate both the innate and adaptive immunity [141]. Galdeano et al. demonstrated that feeding of probiotic fermented milk to the rats increases the number of macrophages and DCs with an increase in IFN-γ, TNF-α, and IL-12 after 5 days of nutrition [142]. Nutrients such as glutamine, arginine, vitamin A, and zinc have protective impacts against enteric infections [143]. Macrophages play an important role in clearance of *Salmonella* in primary infections. Modified arabinoxylan rice bran improves the phagocytic function of macrophages in the in vitro models of RAW264.7 cells [144]. Treatment of macrophages with the modified arabinoxylan rice bran increased the attachment and phagocytosis of yeast cells with an increase in TNF-α and IL-6 [144]. Wang et al. showed an enhanced *Salmonella* specific immune response in the orally vaccinated mice with attenuated *Salmonella* and fed with white button mushroom powder as compared to the only vaccinated mice [145]. The white button mushroom fed mice had higher number of *Salmonella* specific fecal IgA, IFN-γ, and TNF-α in splenocytes. These mice also showed an increased number of DCs and activation marker CD40 in splenocytes as compared to the control mice [145]. These studies show that dietary interventions could modulate pro-inflammatory responses and manipulate the innate and adaptive immunity [141].

## 4. Conclusions

Various dietary components could have considerable efficacy on prevention of *Salmonella* serovars infections. These effects may involve various mechanisms through impacting the gastrointestinal microbiota, immune system, and epithelium. The efficacy of various bioactive compounds for inhibiting the proliferation of *Salmonella* serovars in various in vitro, in vivo, animal models, and randomized studies reviewed creates the opportunity of mitigating the burden of Salmonellosis through dietary intervention. Despite striking similarities, animal models have major differences with human anatomy, as such delineated differences should be considered diligently for interpretation of these studies. Clinical equipoise, cost, time, and other ethical issues are also major curtailments for further conduct of randomized clinical trials with human subjects. The vast majority of the discussed literature demonstrate efficacy and mechanism of action of a sole bioactive compound. Pre-clinical studies investigating synergism and/or antagonistic activities of an array of bioactive compounds, additional randomized clinical trials, and well-planned epidemiological studies with comprehensive plans for control of confounders could augment the development of a validated and evidence-based guideline for mitigating the public health burden of human Salmonellosis through dietary compounds.

## Figures and Tables

**Table 1:** Strengths and weaknesses of *Salmonella* models discussed in the current study.

Infection Model	Strength	Weaknesses
***Salmonella* Culture (Non-Host)**	Direct interaction with the pathogen without confounders	Does not represent the interaction of dietary components with the host
**Co-culture of *Salmonella* with host cell**	Increased complexity of interaction compared to only pathogen culture, represents effects of intervention on the pathogen as well as the host	Does not represent involvement of all the host cell types that simultaneously happen together in human
**Rodent Models**	Represent a complex living system, very economical and convenient, ease in genetic manipulation to know mechanistic pathways	No diarrhea and vomiting, different intestinal immunity, different gastric environment, and anatomical structures
**Pig Models**	Similar to humans in body composition, cardiovascular, renal, nutritional, immunological, metabolic, and gastrointestinal aspects	Different than humans in *Salmonella* colonization pattern, gastric acidity, bile quantities, mucus thickness, immune system, not economical, not convenient
**Calf Models**	Develop similar clinical and pathological features such as diarrhea and enteritis	Stomach structure is different, not economical
**Clinical Trials**	The ideal model	Difficult to study the preventive effects of interventions due to ethical considerations
